# Preparing for participation in the centers for Medicare and Medicaid Services’ bundle care payment initiative—advanced for major bowel surgery

**DOI:** 10.1186/s13741-022-00286-9

**Published:** 2022-12-09

**Authors:** Caitlin R. Collins, Mary Kathryn Abel, Amy Shui, Gina Intinarelli, Julie Ann Sosa, Elizabeth C. Wick

**Affiliations:** 1 Department of Surgery, University of California, San Francisco, 513 Parnassus Avenue, HSW 1601, San Francisco, CA 94143 USA; 2 School of Medicine, University of California, San Francisco, San Francisco, CA USA; 3 Department of Epidemiology and Biostatistics, University of California, San Francisco, San Francisco, CA USA; 4 Office of Population Health, University of California, San Francisco, San Francisco, CA USA

**Keywords:** Alternative payment models, Bundled payment, Surgical risk calculator, Surgical readmissions, Surgical outcomes

## Abstract

**Background:**

As healthcare costs rise, there is an increasing emphasis on alternative payment models to improve care efficiency. The bundled payment represents an alternative reimbursement model gaining popularity within the surgical sphere. We aimed to assess where the largest opportunities for care improvement lay and how best to identify patients at high risk of suffering costly complications.

**Methods:**

We utilized itemized CMS claims data for a retrospective cohort of patients between 2014 and 2016 who met inclusion criteria for the Major Bowel Bundled Payment Program and performed a cost analysis to identify opportunities for improved care efficiency. Based on the results of this cost analysis, we identified readmissions as a target for improvement. We then assessed whether the American College of Surgeons’ National Surgical Quality Improvement Program surgical risk calculator (ACS NSQIP SRC) could accurately identify patients within our bundled payment population who were at high risk of readmission using a logistic regression model.

**Results:**

Our study cohort included 252 patients. Readmissions accounted for 12.8% of the average total care episode cost with a coefficient of variation of 2.72, thereby representing the most substantial, non-fixed cost for our bundled payment patients. Patients readmitted within their 90-day care episode were 2.53 times more likely to be high-cost (>$60,000) than patients not readmitted. However, the ACS NSQIP SRC did not accurately predict patients at high risk of readmission within the first 30 days with an AUROC of 0.58.

**Conclusions:**

Our study highlights the importance of reducing readmissions as a central component of improving care for bowel surgery bundled payment patients. Preventing such readmissions requires accurate identification of patients at high risk of readmission; however, current risk prediction models lack the adaptability necessary for this task.

## Introduction

The annual cost of surgical care in the USA is a staggering $400 billion, and it is projected that surgical expenditures will reach 7.3% of the national gross domestic product by the year 2025 (Muñoz et al., 2010; Gani et al., 2016). Over the past decade, there has been an effort to implement alternative payment models to combat the rising cost of healthcare in the USA. In 2010, the Centers for Medicare and Medicaid Innovation (CMMI) proposed the Bundled Payment for Care Improvement Initiative (BPCI), in which one fixed payment is delivered to cover hospital, professional, and additional services for particular diagnoses or procedures during a single care episode (Bundled Payments for Care Improvement (BPCI); American College of Surgeons). Unlike the current fee-for-service model which lacks incentives for providers to limit costs, this model shifts more financial responsibility for a patient’s total care costs onto hospitals and providers. In the event that a hospital is able to improve the quality and value of care delivered during the 90-day program window following surgery, the savings are shared between the hospital and the Centers for Medicare and Medicaid Services (CMS). Alternatively, if the hospital fails to improve patient care, then the hospital will incur a financial penalty.

Although the program began as a voluntary initiative, the bundled payment model has become mandatory for certain procedures like joint replacements, where the association between BPCI and healthcare improvement has been well studied (Jubelt et al., 2017). Hospitals have had the option to participate in BPCI for major bowel surgery since 2013. However, there are limited reports in the literature about hospitals’ experiences, and several researchers have continued to question the feasibility of bundled payment for major bowel surgery given the heterogeneity and increased complexity of this patient population (Gani et al., 2016; Sibia et al., 2019).

The University of California, San Francisco (UCSF) Health, was one of five large, academic medical centers that elected to participate in the second iteration of the program—BPCI-Advanced (BPCI-A) Major Bowel Bundle Program—which launched at UCSF in October 2018. We elected to participate in the program as a way of preparing for the inevitable changes in surgical care delivery that will be coming over the next decade. As one of our first goals of participation in the bundled payment program, we sought to identify tools to preoperatively risk-stratify our patients in order to effectively direct patient navigation and social services to patients at high risk of utilizing excessive healthcare resources. Due to the wide adoption, repeated validation, and robust nature of the American College of Surgeons’ National Surgical Quality Improvement Program surgical risk calculator (ACS NSQIP SRC), we hypothesized that it could be an effective tool for risk stratification for the UCSF BPCI-A Major Bowel Bundle Program patients (Ingraham et al., 2010; Bilimoria et al., 2013; Jiang et al., 2018; Liu et al., 2016; Lubitz et al., 2017).

## Methods

### Study overview

As part of our planned participation in the major bowel bundled payment program, CMS provided data on a cohort of patients at our institution who were treated between 2014 and 2016 and who would have met the criteria for inclusion in the BPCI-A program. This time period represented our “baseline” performance and was used by CMS to calculate price targets. Based upon these historical costs, the negotiated reimbursement amount from CMS for one care episode in the BPCI-A Major Bowel Bundle Program was set at $60,000. CMS would allocate $60,000 for all of the care required for a single patient’s 90-day care episode, but UCSF would be responsible for the cost of any care delivered over that amount. Any patient with a care episode exceeding $60,000 was defined as “high-cost”. We utilized the itemized claims data to identify areas for improvement prior to program initiation. The BPCI-A program for major bowel includes patients undergoing intra-abdominal operations that involve some element of a bowel resection and fall into the qualifying Medicare Severity Diagnosis Related Groups (MS-DRGs 329, 330, and 331). As such, the patients included in our study underwent either elective or emergent small bowel, colon, and/or rectal procedures for acute conditions (e.g., ischemia, obstruction, or perforation), colorectal cancer, benign disease, and inflammatory bowel disease. All included patients had Medicare as their primary insurer and were enrolled in Medicare Part A & B. Patients who were enrolled in Medicare Advantage were excluded from the CMS program.

CMS provided itemized claims data that included all costs within the 90-day care episode, which began when each patient was admitted for a procedure that fell within a designated DRG. Data included every hospitalization within the 90-day episode, including the “Anchor Stay” (the initial acute care hospitalization) and any readmissions following the Anchor Stay. All claims related to professional fees, outpatient clinic visits, durable medical goods, hospice, pharmacy, rehabilitation or skilled nursing service, and home service (including home health, physical therapy, and ostomy) claims after the index hospitalization were provided. For analysis, contributions from less frequent cost categories were aggregated into an “Other” category and included inpatient psychiatry, inpatient rehabilitation, transfer, physical therapy, and hospice costs.

The preoperative and procedure-related information was abstracted from the electronic health record for each patient and was entered into the ACS NSQIP SRC to determine the predicted risk of readmission. The ACS NSQIP SRC is a well-validated tool used in hospitals around the USA, which utilizes 20 patient variable inputs as well as the planned procedure to predict 30-day outcomes in patients (including readmission risk) following surgery.

This study was deemed exempt by the Institutional Review Board at UCSF.

### Statistical analysis

Itemized claims data were aggregated for the cohort by calculating the mean across each claim category for all patients. The variability of cost within each category was analyzed and compared by calculating the coefficient of variation (c_v_)—a standardized measure of dispersion—for each cost category. The average relative contribution of each cost category was also calculated as a percentage of the average total care episode cost. A cost category was targeted for improvement based on the combination of a high coefficient of variation with a high relative contribution to the total care episode cost. A relative risk was calculated to evaluate whether readmission status affected the likelihood of having a high-cost care episode (defined as >$60,000). A logistic regression model was used to assess the predictive performance of the ACS NSQIP SCR’s anticipated readmission risk with the actual 30-day readmission rate realized in the data. All participants were included in the logistic regression model. The area under the receiver operator curve (AUROC), also known as the c-statistic, was calculated to assess the discrimination ability of the model. All hypothesis tests were two-sided, and the significance threshold was set to 0.05. The statistical analyses were performed using R Studio (RStudio Team, 2019)—an open-access programming platform for statistical computing.

## Results

### Population demographics and comorbidities

Our study cohort included 252 patients treated at UCSF between 2014 and 2016 who met the inclusion criteria for the Major Bowel Bundled Payment Program (Table 1). The mean patient age was 70.1 years, and 54% (*n* = 137) of the cohort were female. Approximately, 56.7% (*n*=143) of patients were designated as the American Society of Anesthesiologists (ASA) class 3 at the time of their operation. An emergency procedure was performed for 13.9% (*n*=35) of patients. The surgical approach was open for 43.7% of procedures. The patient population was complex, with 50.8% (*n*=128) carrying a diagnosis of hypertension requiring medication and 20.2% (*n*=51) with diabetes (both insulin- and non-insulin-dependent). Nearly 20% (*n*=50) of patients had disseminated cancer at the time of surgery. The median length of stay was 6 days (range: 0–39 days), and the 90-day readmission cumulative incidence rate was 36.5%.

**Table 1:** Patient demographics and comorbidities

	Mean (SD*)	**Range**
Age (years)	70.1 (11.1)	27–98
BMI	25.6 (6.38)	14.7–50.0
	***N*** **(total = 150)**	**Percent (%)**
Gender		
Female	137	54.4
Male	115	45.6
Functional status		
Independent	240	95.2
Partially dependent	7	2.8
Totally dependent	5	2
ASA class		
ASA 1	2	0.8
ASA 2	98	38.9
ASA 3	143	56.7
ASA 4	9	3.6
ASA 5	0	0
Emergency case		
No	217	86.1
Yes	35	13.9
Hypertension requiring medication		
No	124	49.2
Yes	128	50.8
Diabetes		
No	201	79.8
Non-insulin dependent	40	15.9
Insulin dependent	11	4.3
Disseminated cancer		
No	202	80.2
Yes	50	19.8
Steroid use		
No	226	89.7
Yes	26	10.3
History of severe COPD^†^		
No	234	92.9
Yes	18	7.1
Dyspnea		
No	233	92.5
Moderate exertion	18	7.1
At rest	1	0.4
Smoker within 1 year		
No	241	95.6
Yes	11	4.4
Ascites		
No	247	98
Yes	5	2
Sepsis within 48 h		
No	245	97.2
Yes	7	2.8
CHF^‡^ exacerbation or diagnosis within 30 days		
No	250	99.2
Yes	2	0.8
Ventilator dependence		
No	252	100
Yes	0	0
Acute renal failure		
No	252	100
Yes	0	0
Dialysis		
No	252	100
Yes	0	0

*SD* standard deviation, *COPD* chronic obstructive pulmonary disease, *CHF* congestive heart failure

### Care episode claims analysis

**Fig. 1:**
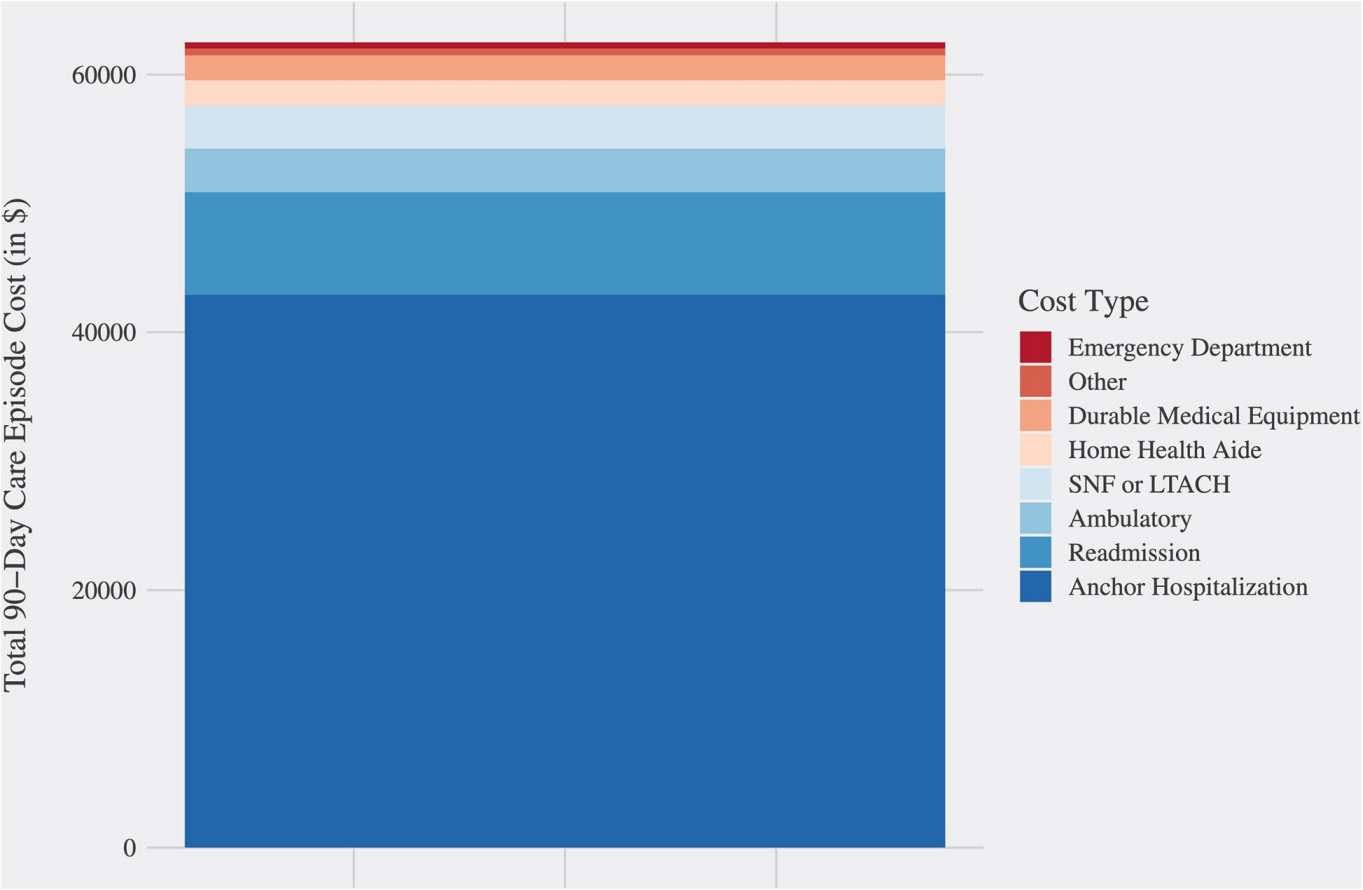
Average total care episode cost color-coded by cost type

The itemized CMS claims data for all patients in the bundled payment cohort were averaged across each cost category (Fig. 1), and their respective coefficient of variation was calculated. For reference, the c_v_ ranged from 0.73 to 5.05. The combined anchor stay and professional fee (physician services) claims accounted for 68.8% of the average care episode cost. The c_v_ for these payments was 0.73, suggesting a relatively fixed payment amount across patients. Readmission payments represented the next highest cost category at 12.8% of the average care episode cost but demonstrated high variability across patients (c_v_ = 2.72). Although the c_v_ was higher for the durable medical equipment (c_v_ = 4.03) and the “other” (c_v_ = 5.05) cost categories, they both represented a relatively small overall component of the total care episode costs on average (3.1% and 0.8%, respectively). Given both the high coefficient of variation and the significant contribution to the average total care episode cost, we identified readmissions as a prime target for intervention to improve care efficiency for BPCI-A patients.

### Readmissions

**Fig. 2:**
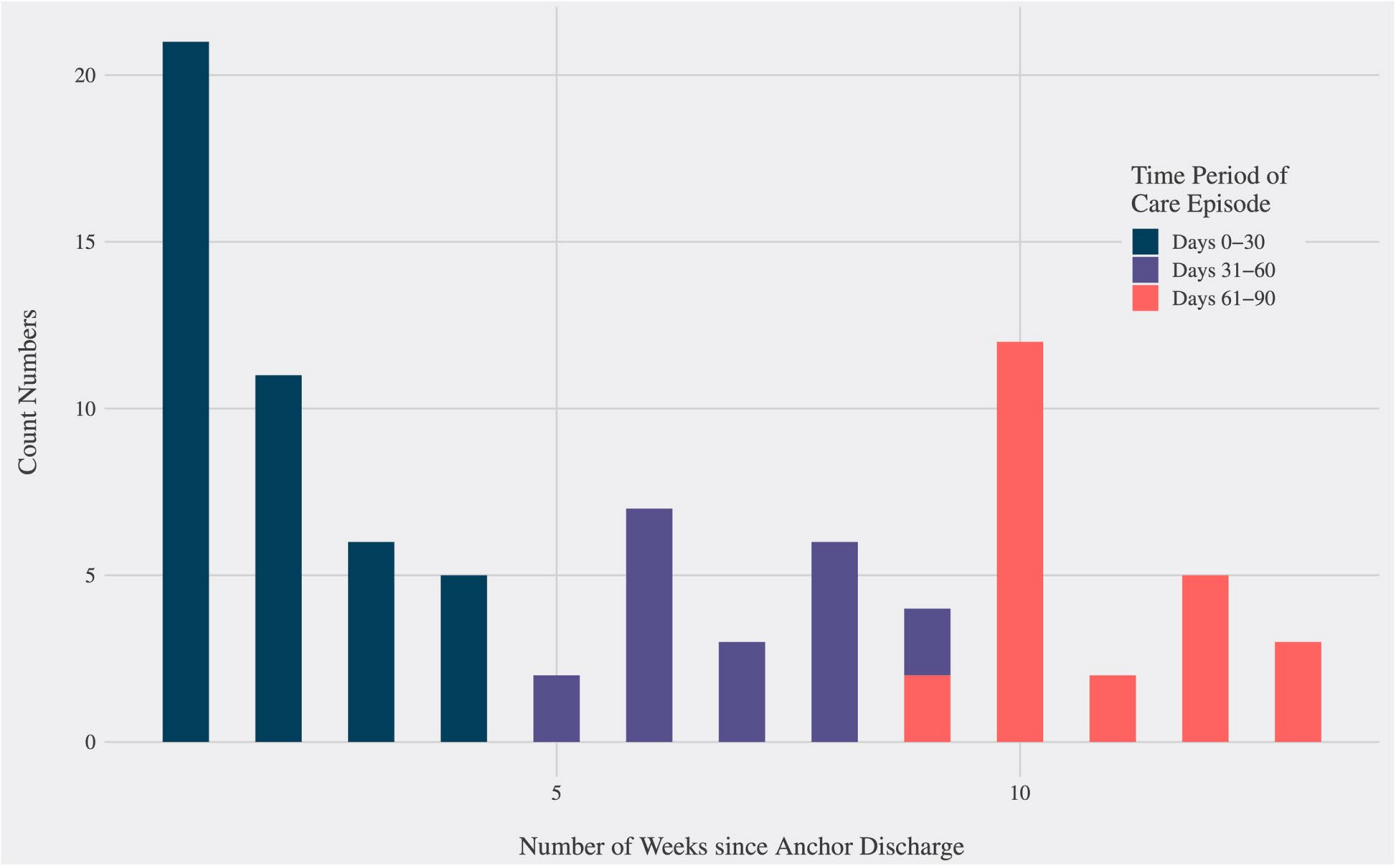
Histogram of readmission time course

When looking closer at readmissions across the 90-day timeline, the first week following discharge from the hospital represented the highest risk time for readmission in the care episode. If a patient was readmitted, there was a 24% chance that the readmission would occur in the first week after discharge from the hospital. Overall, 49.4% (*n*=43) of all readmissions to the hospital occurred in the first 30 days of the 90-day care episode (Fig. 2). The other half of the readmissions were evenly distributed across the remaining 31–90 days of the care episode. Patients who were readmitted during their 90-day care episode were more likely to subsequently have a high-cost total care episode (defined as an episode over the pre-defined target of $60,000 agreed upon by CMS and UCSF) when compared to individuals who were not readmitted (RR = 2.53, 95% CI 1.86–3.43, *p* < 0.001) (Table 2). With each additional readmission during the care episode, the likelihood that the patient would fall within the high-cost category also increased (Table 3).

**Table 2:** Ninety-day readmissions are significantly associated with high-cost outcomes in major bowel-bundled payment patients

90-day readmission status		**Cost**		**Total**
		**Low**	High*	
	**Not readmitted**	138	47	**185**
	**Readmitted**	24	43	**67**
	**Total**	**162**	**90**	**252**
	*High cost—total care episode cost exceeding $60,000 (in US dollars)			
	**Relative risk ratio**	**95% CI**	***P*** **value**	
	2.53	(1.86–3.43)	<0.001

**Table 3:** Total care episode costs progressively increase with each additional 90-day readmission in major bowel-bundled payment patients

Number of readmissions in a 90-day episode	Percentage of patients in high-cost* category
0	25.40%
1	54.20%
2	84.60%
3	100%

*High-cost—total care episode cost exceeding $60,000 (in US dollars)

### ACS NSQIP surgical risk calculator evaluation

**Fig. 3:**
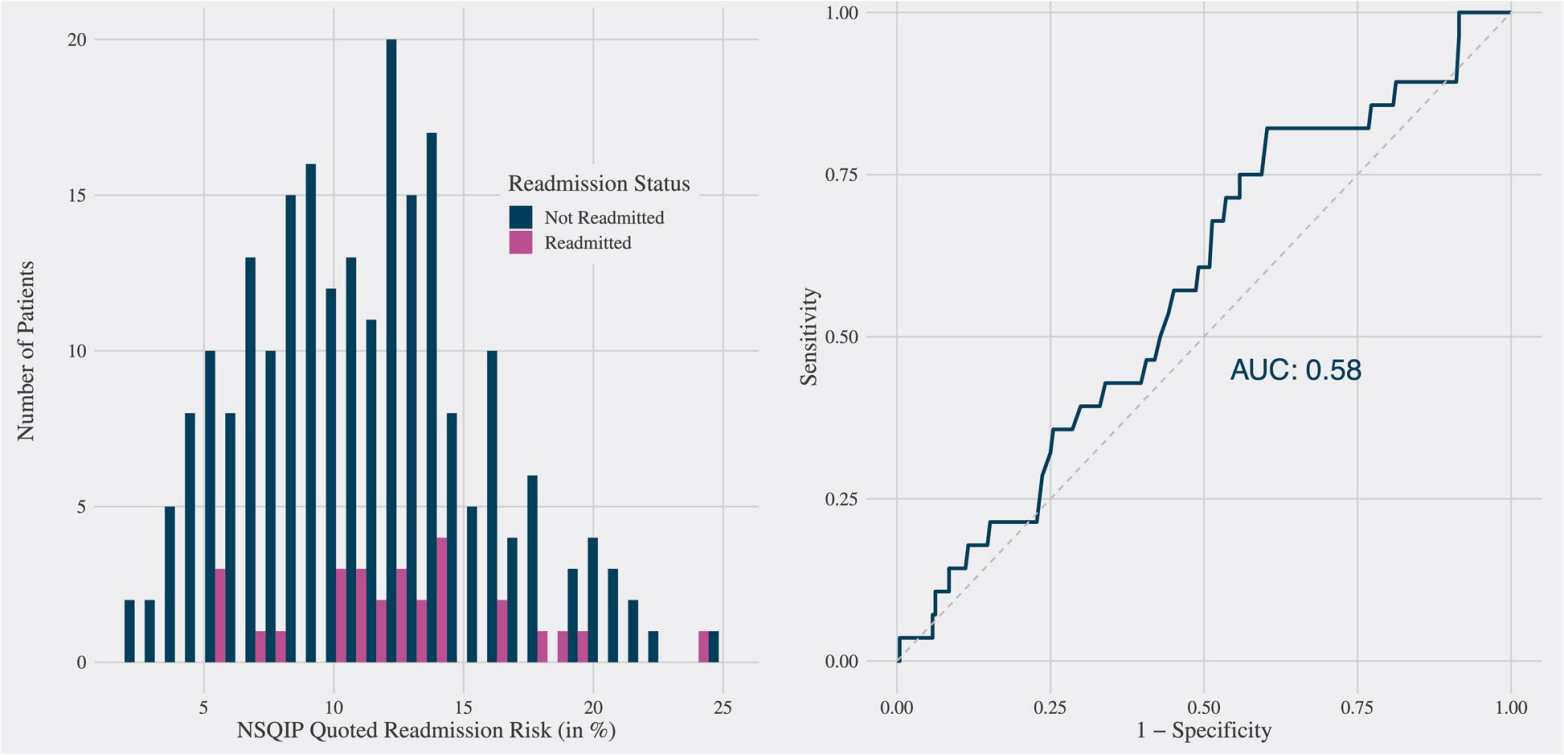
**a** Distribution of NSQIP-calculated readmission risk scores color-coded by a true 30-day readmission status. **b** ROC curve for readmission risk score

Recognizing that readmissions would be an essential target for improvement to succeed in the program, the ACS NSQIP SRC 30-day readmission risk was compared with actual hospital readmissions for our cohort of patients using logistic regression. The overlapping distribution of SRC readmission risk scores between patients who were readmitted compared to those who were not readmitted demonstrates the poor discriminative capability of the SRC for readmission in this cohort (Fig. 3a). The resulting AUROC was 0.58, suggesting that the ACS NSQIP SRC predicted readmission only slightly better than chance (Fig. 3b).

## Discussion

As healthcare spending continues to rise in the USA, there is a strong push to mitigate healthcare costs and incentivize quality and efficiency by moving away from the classic fee-for-service model. The CMS BPCI-A alternative payment model represents an innovative approach to payment restructuring that may be necessary to meet the rising cost of healthcare. Under the BPCI model, all episodes of care have a fixed cost, with both savings and penalties placed on the hospital (Bundled Payments for Care Improvement (BPCI), 2022). However, surgery is nuanced, and patient needs and healthcare utilization can vary widely from one procedural area to another. Our analysis demonstrates that the major driver of 90-day healthcare utilization and costs in major bowel bundle patients is readmission at an acute care hospital during the bundle window. In contrast, the published experiences with comprehensive joint replacement found that much of the utilization was related to the use of inpatient rehabilitation after discharge from an acute hospital (Glickman et al., 2018; Dummit et al., 2016; Finkelstein et al., 2018; Tsai & Miller, 2015).

Based on our analysis of the major bowel bundled payment cohort, the greatest opportunity for improvement would be to reduce readmissions by accurately identifying patients who were high risk for readmission and thus more likely to have high-cost care episodes. The ACS NSQIP SCR is a well-validated and widely available tool that has been studied in a number of broad surgical scenarios to predict readmission (Glance et al., 2014; Wang et al., 2017). However, the calculator was unable to predict readmission risk or total care episode cost in our bundled payment patient cohort. There are several factors that may account for this failure. While the risk calculator was calibrated and validated for patients encompassing a wide range of ages, co-morbidities, and surgical conditions, our population was more complex and heterogeneous than the population utilized for the calculator (Cohen et al., 2009; Cohen et al., 2017). In particular, our major bowel bundle surgical cohort was older, had higher ASA scores, higher rates of emergency surgery, and were more likely to have disseminated cancer compared to the ACS NSQIP population of colorectal surgery patients (FastStats). The ACS NSQIP SRC might be useful for counseling a patient about their risk of various outcomes based on their co-morbidities when compared to the average NSQIP patient at the average NSQIP-affiliated hospital, but it lacks the specificity and modifiability needed to risk stratify patients within specific contexts—such as single-institution bundled payment programs (Sahara et al., 2020).

An important caveat to the use of risk calculators concerns *how* these scores are used. While a score that quantifies the possibility of increased risk and cost would ideally be used to direct appropriate care and services to these patients, it also could be used to limit access to surgical care over the concern for penalties in a bundled payment model, as suggested by Tsai et. al (Tsai & Miller, 2015). It is essential that appropriate risk adjustment techniques be employed to adjust for utilization of both in-hospital and post-discharge resources by high-risk patients. This recommendation is in line with the “Whole Person Care” approach that has been adopted by Medi-Cal, which might elucidate additional strategies for the management of patients who are more likely to incur high healthcare costs (Whole person care pilots: department of health care services; Lucy Pagel & Haycock, 2019). Assessing the impact of better integration and coordination of medical, behavioral, and social services to address the needs of high-cost patients warrants further study.

There are a number of limitations in the current study that are the result of retrospective data analysis. Miscoding or under-coding of medical conditions and complications may have occurred during the process of administrative data collection. Inaccurate or absent documentation within the electronic medical record also remains a concern. In the end, our analysis was performed on a unique but relatively small cohort, which increases the possibility for sampling bias and limits statistical power and generalizability. However, given the dearth of actual hospital experience with the Major Bowel Bundled Payment program and the accelerated integration of alternative payment models in the surgical landscape, the findings highlight the need for better study of the CMS bundle eligible general surgery patients with an emphasis on the development of predictive risk models. Such models will be essential for hospitals to succeed in developing targeted interventions for those patients most in need of additional support for their transition from acute hospitalization back to the community.

## Conclusion

Many of the alternative payment models, including bundled payment, compel providers to consider all of the facets that contribute to the severity and complexity of the disease. The change in reimbursement-related incentives will require a concomitant paradigm shift in how surgeons approach the care of their patients, including those at risk of incurring high healthcare costs. With 90-day-long care episodes, adequate long-term support of patients’ chronic medical conditions and the home environment becomes just as important as excellent peri-operative care. CMS and other payors have committed to evolve reimbursement strategies, and surgeons need to be engaged in participating in alternative payment models and stringently evaluating their feasibility. While the SRC is an effective tool for counseling patients about their expected course after surgery, it is not effective in predicting outcomes in the select BPCI-A major bowel surgery population. We urgently need more sophisticated and adaptive risk stratification tools to improve care efficiency and survive within a value-based payment structure.

## Data Availability

The datasets used and/or analyzed during the current study are available from the corresponding author on reasonable request.

## Abbreviations

CMMI: Centers for Medicare and Medicaid Innovation
BPCI: Bundled Payment for Care Improvement Initiative
CMS: Medicare and Medicaid Services
UCSF: University of California, San Francisco
BPCI-A: Bundled Payment for Care Improvement Initiative-Advanced
ASC NSQIP SRC: American College of Surgeons’ National Surgical Quality Improvement Program surgical risk calculator
MS-DRG or DRG: Medicare Severity Diagnosis Related Groups
AUROC: Area under the receiver operator curve
ASA: American Society of Anesthesiologists
